# Functional assessment of the glycoproteins of a novel Hendra virus variant reveals contrasting fusogenic capacities of the receptor-binding and fusion glycoproteins

**DOI:** 10.1128/mbio.03482-23

**Published:** 2024-12-20

**Authors:** Andrew Z. Ma, Yao Yu Yeo, Jean F. Lee, Colin M. Kim, Shahrzad Ezzatpour, Carolina Menchaca, Viraj Upadhye, Edward J. Annand, John-Sebastian Eden, Raina K. Plowright, Alison J. Peel, David W. Buchholz, Hector C. Aguilar

**Affiliations:** 1 Department of Microbiology and Immunology, College of Veterinary Medicine, Cornell University, Ithaca, New York, USA; 2 Epidemiology Surveillance and Laboratory Section, Animal Health Policy Branch, Animal Division, Department of Agriculture Fisheries and Forestry, Canberra, Australian Capital Territory, Australia; 3 Westmead Institute for Medical Research, Centre for Virus Research, Westmead, New South Wales, Australia; 4 Department of Public and Ecosystem Health, College of Veterinary Medicine, Cornell University, Ithaca, New York, USA; 5 Centre for Planetary Health and Food Security, School of Environment and Science, Griffith University, Nathan, Queensland, Australia; University of Pennsylvania, Philadelphia, Pennsylvania, USA; The Ohio State University, Columbus, Ohio, USA

**Keywords:** Hendra virus, receptor-binding protein, fusion protein, receptor, syncytia, *Henipavirus*, paramyxovirus

## Abstract

A novel Hendra virus (HeV) genotype (HeV genotype 2 [HeV-g2]) was recently isolated from a deceased horse, revealing high-sequence conservation and antigenic similarities with the prototypic strain, HeV-g1. As the receptor-binding (G) and fusion (F) glycoproteins of HeV are essential for mediating viral entry, functional characterization of emerging HeV genotypic variants is key to understanding viral entry mechanisms and broader virus-host co-evolution. We first confirmed that HeV-g2 and HeV-g1 glycoproteins share a close phylogenetic relationship, underscoring HeV-g2’s relevance to global health. Our *in vitro* data showed that HeV-g2 glycoproteins induced cell-cell fusion in human cells, shared receptor tropism with HeV-g1, and cross-reacted with antibodies raised against HeV-g1. Despite these similarities, HeV-g2 glycoproteins yielded reduced syncytia formation compared to HeV-g1. By expressing heterotypic combinations of HeV-g2, HeV-g1, and Nipah virus (NiV) glycoproteins, we found that while HeV-g2 G had strong fusion-promoting abilities, HeV-g2 F consistently displayed hypofusogenic properties. These fusion phenotypes were more closely associated with those observed in the related NiV. Further investigation using HeV-g1 and HeV-g2 glycoprotein chimeras revealed that multiple domains may play roles in modulating these fusion phenotypes. Altogether, our findings may establish intrinsic fusogenic capacities of viral glycoproteins as a potential driver behind the emergence of new henipaviral variants.

**IMPORTANCE:**

HeV is a zoonotic pathogen that causes severe disease across various mammalian hosts, including horses and humans. The identification of unrecognized HeV variants, such as HeV-g2, highlights the need to investigate mechanisms that may drive their evolution, transmission, and pathogenicity. Our study reveals that HeV-g2 and HeV-g1 glycoproteins are highly conserved in identity, function, and receptor tropism, yet they differ in their abilities to induce the formation of multinucleated cells (syncytia), which is a potential marker of viral pathogenesis. By using heterotypic combinations of HeV-g2 with either HeV-g1 or NiV glycoproteins, as well as chimeric HeV-g1/HeV-g2 glycoproteins, we demonstrate that the differences in syncytial formation can be attributed to the intrinsic fusogenic capacities of each glycoprotein. Our data indicate that HeV-g2 glycoproteins have fusion phenotypes closely related to those of NiV and that fusion promotion may be a crucial factor driving the emergence of new henipaviral variants.

## INTRODUCTION

Hendra virus (HeV) is a prototypical member of the *Henipavirus* (HNV) genus in the *Paramyxoviridae* family of enveloped, negative sense, single-stranded RNA viruses. HNVs consist of emerging zoonotic viruses geographically distributed across Australia, Asia, and Africa, which includes Nipah virus (NiV), Cedar virus (CedV), and Mojiang virus (MojV) (1–3), and others. HeV was first detected in 1994 in Brisbane, Queensland, Australia, as a cause of severe or fatal illnesses in humans and horses (4). Since then, there have been four fatalities of seven recognized human cases (57% mortality), and HeV continues to cause recurring deadly infections in horses across broad geographical regions of Australia (5). The main symptoms of HeV infections in horses and humans include acute encephalitis and respiratory distress, although bats show no overt signs of disease (5, 6). Australian *Pteropus* spp. or fruit bats, colloquially known as flying foxes, are hosts for HeV (6), and HeV transmissions have occurred from bats to horses, between horses, horses to humans, and horses to dogs (5). Experimental studies have also established a broad HeV mammalian host range (6), and the lethality and zoonotic ability of HeV have rendered it a biosafety level-4 select agent that poses substantial threat to human and animal health (7).

HNVs contain two glycoproteins, the receptor-binding protein or attachment (G) and fusion (F) glycoproteins, that cooperatively mediate membrane fusion and viral entry at the virus-cell and cell-cell interfaces, the latter resulting in the formation of multinucleated syncytial cells which are prognostic features of both *in vitro* and *in vivo* infections (8). HNV G contains the receptor-binding domain and utilizes ephrin ligands as entry receptors (9–15); MojV G and LayV G entry receptors are still unknown and are unlikely to be ephrins (16–18). As ephrins are relatively conserved across mammalian species, the broad mammalian host tropism of HNVs is attributed to the use of ephrins as entry receptors (8, 19). HNV F contains the fusion peptide in its F_1_ region, which is exposed after proteolytic activation of immature F_0_ into mature F_1_/F_2_ subunits (20, 21). Upon receptor binding, HNV G undergoes a series of conformational changes that trigger proteolytically activated HNV F, which leads to HNV F-induced membrane fusion (22). Besides the indispensable roles of HNV G and F in viral entry, they are also the main immunogenic targets and can elicit protective humoral immunity against lethal infections in vaccinated animal models (23).

In 2021, a novel genotype of HeV genotype 2 (HeV-g2) was reported by two independent teams, one isolated from a fatally diseased horse (24) and another detected by PCR in gray-headed flying fox (*Pteropus poliocephalus*) and little red flying fox (*Pteropus scapulatus*) tissue samples (25). HeV-g2 was also subsequently detected in urine from black flying foxes (*Pteropus alecto*) and gray-headed flying foxes, as well as a second fatal equine case (26, 27). The novelty of HeV-g2 was determined via phylogenetic analyses of its genome and the nucleocapsid (N) and polymerase (L) genes, which unequivocally placed HeV-g2 in a separate lineage within the HeV cluster when referenced against other paramyxoviruses (24, 25). The detection of a novel HeV variant that can cause fatal illness reinforces the ongoing risk of natural HeV spillover from flying foxes into susceptible host populations across their broad distribution (24–28). Henceforth, in this study, we refer to the first variant of HeV from 1994 as HeV-g1.

Given the significance of HeV-g1 as a highly pathogenic HNV (7) and the need to understand the molecular mechanisms of HNV glycoproteins, we conducted a phylogenetic and molecular functional analysis of HeV-g2 glycoproteins. Our findings revealed that HeV-g2 has the molecular requirements to infect human cells, that each HeV-g2 glycoprotein exhibits divergent cell-cell fusion phenotypes from HeV-g1, and that the HeV-g2 fusion phenotypes appear more functionally related to those of the NiV glycoproteins. These results further suggest that the intrinsic fusogenic capacities of each glycoprotein may be a potential driver of HeV evolution.

## RESULTS

### HeV-g2 glycoproteins share high identity with HeV-g1

**Fig 1:**
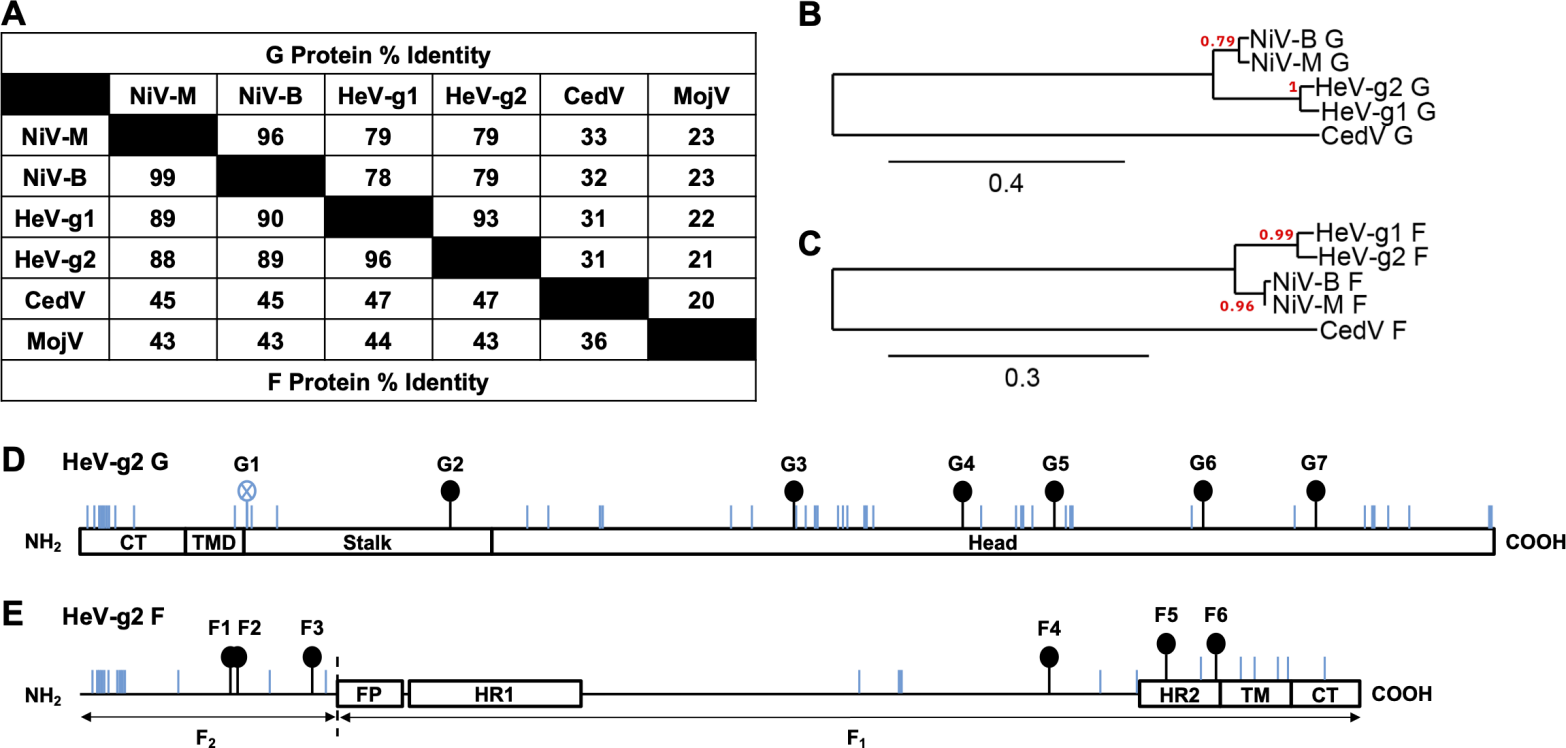
Protein sequence alignments between HeV-g2 and other HNV glycoproteins. (**A**) Percent identities among glycoprotein amino acid sequences of two NiV strains (Malaysian and Bangladesh isolates), two HeV strains, and two other HNVs, CedV and MojV. (**B and C**) Phylogenetic trees comparing glycoprotein amino acid sequences of representative strains from NiV and HeV outbreaks. CedV glycoproteins serve as the outgroup. Branch support values are written in red font and located next to each node. (**D and E**) Schematics of HeV-g2 G and F proteins. The small blue vertical lines indicate mutated residues as compared to HeV-g1. Sticks with filled circles indicate potential N-X-S/T N-glycosylation sites, and sticks with open circles indicate a loss of potential N-X-S/T N-glycosylation sites as compared to HeV-g1.

Since paramyxoviral N and L protein sequences are relatively conserved, they are often used for phylogenetic analyses and assignment of novel paramyxoviruses, although a caveat is the inability of such phylograms to accurately portray zoonotic potential and/or antigenic diversification of novel, emerging viruses (19). Therefore, to assess the relationships between HeV-g2 and HeV-g1 glycoproteins, we first performed an amino acid alignment across their glycoprotein sequences and included other related HNVs: NiV, CedV, and MojV. As expected, we found that HeV-g2 G and F share the highest percent identity with their HeV-g1 counterparts: 93% and 96%, respectively (Fig. 1A). As HeV-g2 G and F also share considerable percent identities with NiV G (79%) and F (88%–89%), we then performed phylogenetic analyses of HeV-g2 glycoprotein sequences against those of representative NiV and HeV outbreak strains to determine if they are indeed most related to HeV-g1 G and F (Fig. 1B and C). Our results affirm this close relationship and allow us to map the domains of HeV-g2 G and F by sequence homology to HeV-g1 G and F (Fig. 1D and E). HeV-g2 G has 603 amino acids, which is one less than HeV-g1 G, due to the loss of an extra methionine at the first residue, while most mutations are found distributed throughout the receptor-binding head domain and at the N-terminus of the cytoplasmic tail (CT) domain (Fig. 1D). HeV-g2 F and HeV-g1 F both have 546 amino acids, with most mutations accumulating at the N-terminus of the F_2_ fragment (Fig. 1E).

### HeV-g1 and HeV-g2 G and F share similar oligomerization, receptor tropism, and antigenic profiles

**Fig 2:**
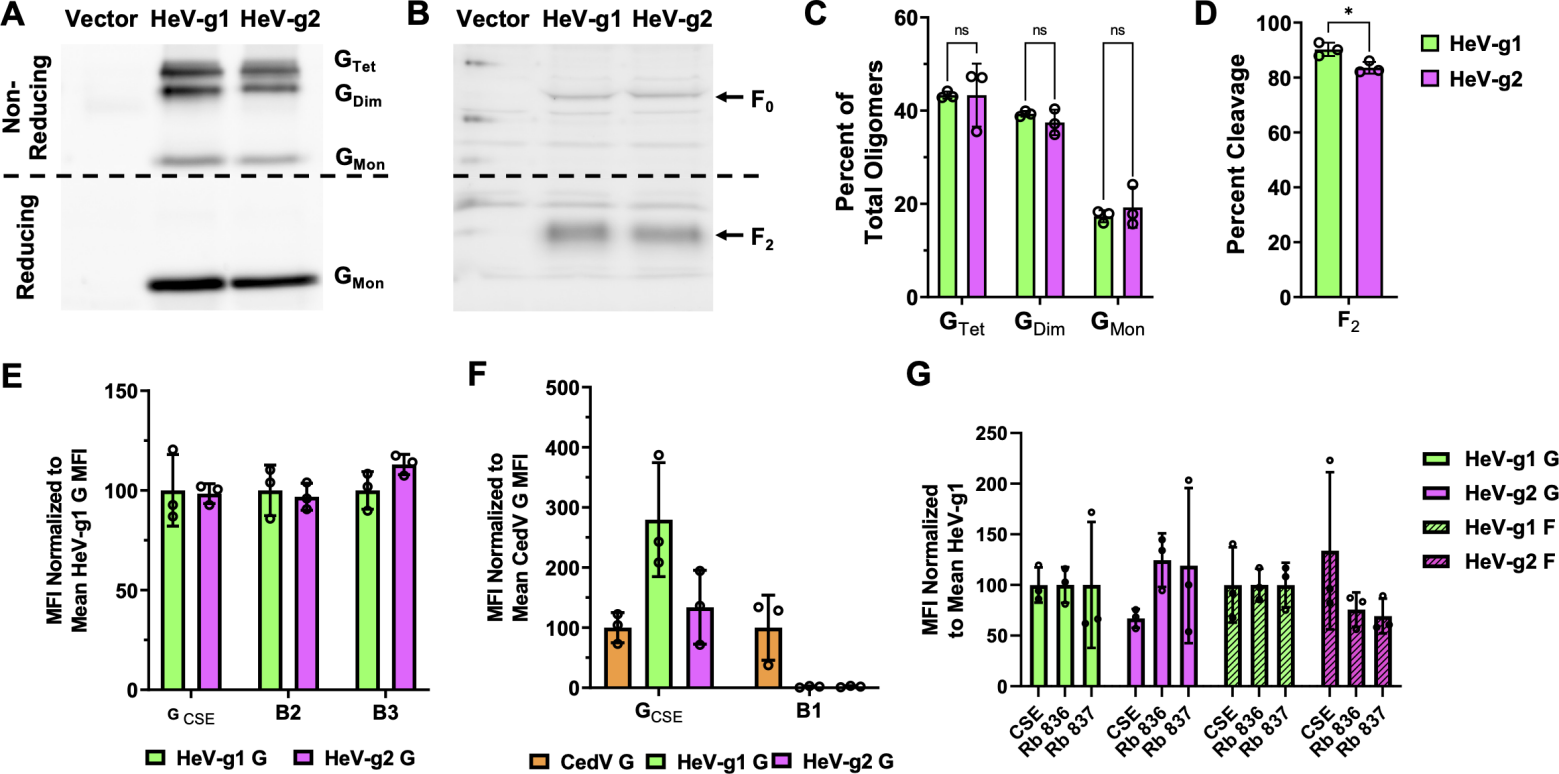
Oligomeric states, receptor tropism, and antigenic profiles of HeV-g1 and HeV-g2 G and F. (**A**) Representative Western blot analysis of HeV-g1 G and HeV-g2 G in non-reducing and reducing conditions. G tetramers (G_Tet_), dimers (G_Dim_), and monomers (G_Mon_) were observed in the non-reducing gel blot. (**B**) Representative Western blot of HeV-g1 and HeV-g2 F proteins in a reducing gel with F_0_ and F_2_ bands indicated. (**C**) Densitometric analysis of G_Tet_, G_Dim_, and G_Mon_ bands (from panel A) as percentages of total G = G_Tet_ + _GDim_ + G_Mon_. (**D**) Analysis of F_2_ bands (from panel B) as percentages of total F = F_0_ + F_2_. (**E**) Relative levels of G cell surface expression (G_CSE_) measured via flow cytometry as the mean fluorescence intensity (MFI) was normalized to HeV-g1 G_CSE_. Relative levels of ephrinB2 or B3 binding were first normalized to the levels of G_CSE_ of the respective HeV and then normalized to the mean HeV-g1 ephrin binding. (**F**) Relative levels of G_CSE_ were first normalized to the mean CedV G_CSE_, set to 100%. Relative levels of ephrinB1 binding were first normalized to the G_CSE_ of the respective HNV and then normalized to the mean CedV ephrinB1 binding. (**G**) Polyclonal anti-HeV-g1 sera taken from two different rabbits (Rb 836 and 837) immunized with HeV-g1 F and G DNA expression plasmids cross-react with HeV-g2 F and G. Relative G_CSE_ and F_CSE_ were normalized to mean HeV-g1 G_CSE_ and mean F_CSE_ levels (set to 100%), respectively. Relative levels of serum binding were first normalized to CSE of the respective protein and then normalized to mean HeV-g1 serum binding. Statistical significance was determined via one-way analysis of variance with Tukey’s multiple comparison test. A *P* value of >0.05 is indicated as ns, and other *P* values are given the following indicators: *P ≤ 0.05, **P ≤ 0.01, ***P ≤ 0.001, ****P ≤ 0.0001. ns, non-significant.

To assess the relevance of HeV-g2 glycoproteins in human cells, we sought to determine if the proteins of HeV-g2 G and F (24, 25) are functional and can mediate viral entry in human cells *in vitro*. We first cloned codon-optimized HeV-g2 sequences into mammalian expression vectors and attached a C-terminus HA tag for HeV-g2 G and an internal FLAG tag for HeV-g2 F, as previously published (29–31). As only tetrameric HNV G and cleaved HNV F are functional (20, 21, 32, 33), we verified oligomerization of HeV-g2 G (Fig. 2A) and cleavage of HeV-g2 F (Fig. 2B) in cell culture by transfecting individual G or F plasmids into HEK 293T cells and immunoblotting cell lysates under reducing or non-reducing conditions. We found that the strength of oligomerization of HeV-g2 G is consistent with that of HeV-g1 G (Fig. 2C). However, we observed a small but statistically significant difference in the cleavage of F, with HeV-g2 showing ~5% less cleaved F than HeV-g1 (Fig. 2D).

Different HNVs can use different receptors for entry into host cells; for instance, HeV-g1 utilizes ephrinB2 and B3, but not B1 (10–12); CedV utilizes ephrinB1 and B2, but not B3 (14, 15); and GhV utilizes ephrinB2, but not B1 or B3 (13). Therefore, we sought to assess HeV-g2’s receptor tropism in comparison to HeV-g1 and first tested whether HeV-g2 G could bind soluble ephrinB2 or B3. HEK 293T cells transfected to express HeV G at similar cell surface expression (CSE) levels demonstrated that HeV-g2 and HeV-g1 G bound comparable amounts of ephrinB2 and B3 (Fig. 2E). Furthermore, because CedV G uses ephrinB1 for entry, while HeV-g1 does not, we further tested and found that HeV-g2 G was also unable to bind soluble ephrinB1 (Fig. 2F). These results indicate that as with HeV-g1, HeV-g2 utilizes ephrinB2 and B3, but not B1, as viral entry receptors, as expected, given the close phylogenetic relationship between HeV-g2 and HeV-g1. Furthermore, other studies have indicated that HeV-g1 and HeV-g2 G glycoproteins do not differ in their affinity to human ephrinB2 or B3 receptors (28).

To evaluate antigenic similarity between HeV-g2 and HeV-g1, we transfected HEK 293T cells to express individual HeV-g1 or HeV-g2 glycoproteins and found that rabbit anti-HeV-g1 G and F sera (34) cross-reacted with both HeV-g2 G and F (Fig. 2G). This reveals that HeV-g2 glycoproteins may share similar antigenic profiles with HeV-g1 glycoproteins, in alignment with previous studies (24, 28).

### HeV-g2 exhibits less fusogenic activity than HeV-g1, and combinations of HeV-g1 and HeV-g2 glycoproteins yield drastically different cell-cell fusion phenotypes

Given the ability for HeV-g2 glycoproteins to be functionally active in human cells and the relevance of syncytia formation in HNV pathogenesis (8), we compared the ability to form syncytia between HeV-g2 and HeV-g1 glycoproteins. We first optimized transfections of HeV-g2 and HeV-g1 glycoproteins in HEK 293T cells to yield comparable CSE of G and F (G_CSE_ and F_CSE_) and then quantified the number of nuclei inside the syncytia (defined as ≥4 nuclei in each cell) as a previously established method to quantify cell-cell fusion (22, 32, 33). Furthermore, we sought to perform a robust analysis of HeV-g2 glycoproteins’ fusogenic capacities relative to those of HeV-g1. We used a similar method as previously established (30, 31), in which we calculated each genotype’s fusion indices, to account for the effects of CSE on the levels of cell-cell fusion. Fusion indices were obtained by quantifying cell-cell fusion levels and normalizing them to the cell surface expression levels of G (FI_G_) or F (FI_F_).

**Fig 3:**
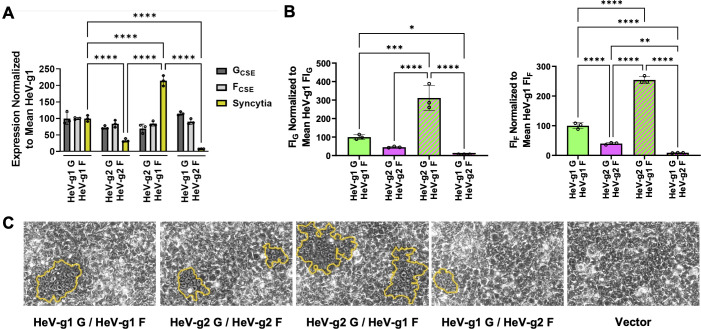
Cell-cell fusion phenotypes from homotypic and heterotypic combinations of HeV-g1 and HeV-g2 glycoproteins. (**A**) Relative levels of G cell surface expression (G_CSE_), F_CSE_, and syncytia counts normalized to their respective HeV-g1 values. (**B**) Fusion indices (FI_G_ and FI_F_) normalized to the mean HeV-g1 FI levels set to 100%. Fusion indices were calculated by dividing syncytia counts by the respective G_CSE_ (FI_G_) or F_CSE_ (FI_F_) levels and then normalizing the fusion indices to the mean HeV-g1 fusion indices set to 100%. (**C**) Representative syncytia images in HEK 293T cells. Statistical significance was determined via one-way analysis of variance with Tukey’s multiple comparison test. Only significant *P* values are shown with the following indicators: *P ≤ 0.05, **P ≤ 0.01, ***P ≤ 0.001, ****P ≤ 0.0001.

We found that at comparable G_CSE_ and F_CSE_ levels, HeV-g2 exhibited less fusogenic activity than HeV-g1 (Fig. 3A), retaining only ~40% to 45% of the level of fusogenicity as indicated in the significantly lower FI_G_ and FI_F_ (Fig. 3B). To further elucidate the mechanisms underlying HeV-g2’s hypofusogenic phenotype, we performed syncytia assay while crossing HeV-g2 and HeV-g1 glycoproteins. Surprisingly, at comparable G_CSE_ and F_CSE_ levels, the HeV-g2 G/HeV-g1 F combination yielded the most fusogenic phenotype, while the HeV-g1 G/HeV-g2 F combination yielded the least fusogenic phenotype (Fig. 3A), as reflected in their significantly higher (FI_G_ ~300%, FI_F_ ~250%) and lower (FI_G_ ~7%, FI_F_ ~9%) respective fusion indices (Fig. 3B). Representative images of syncytia formation levels between homotypic and heterotypic combinations of HeV-g2 and HeV-g1 glycoproteins are shown (Fig. 3C). This suggests that each HNV glycoprotein has a different intrinsic capacity for inducing cell-cell fusion, where HeV-g2 G intrinsically exhibits higher fusion-promoting activity than HeV-g1 G, and HeV-g2 F intrinsically exhibits lower fusogenic activity than HeV-g1 F. Importantly, our findings illustrate a clear functional deviation between HeV-g2 and HeV-g1 glycoproteins despite their high-sequence similarities (Fig. 1A) and conserved receptor tropism (Fig. 2E and F).

### Heterotypic combinations of HNV glycoproteins highlight different intrinsic fusion-promotion capacities for each glycoprotein

After observing divergent fusogenic phenotypes in individual HeV glycoproteins, we sought to examine whether those fusogenic phenotypes would hold in the context of the glycoproteins of a different HNV, NiV. We first optimized the cell surface expressions of each G and F protein from HeV-g1, HeV-g2, and NiV to be at similar levels and then co-expressed all possible pairs of the HNV glycoproteins. Syncytial nuclei were then counted and normalized to G_CSE_ or F_CSE_ levels to compute FI_G_ or FI_F_.

**Fig 4:**
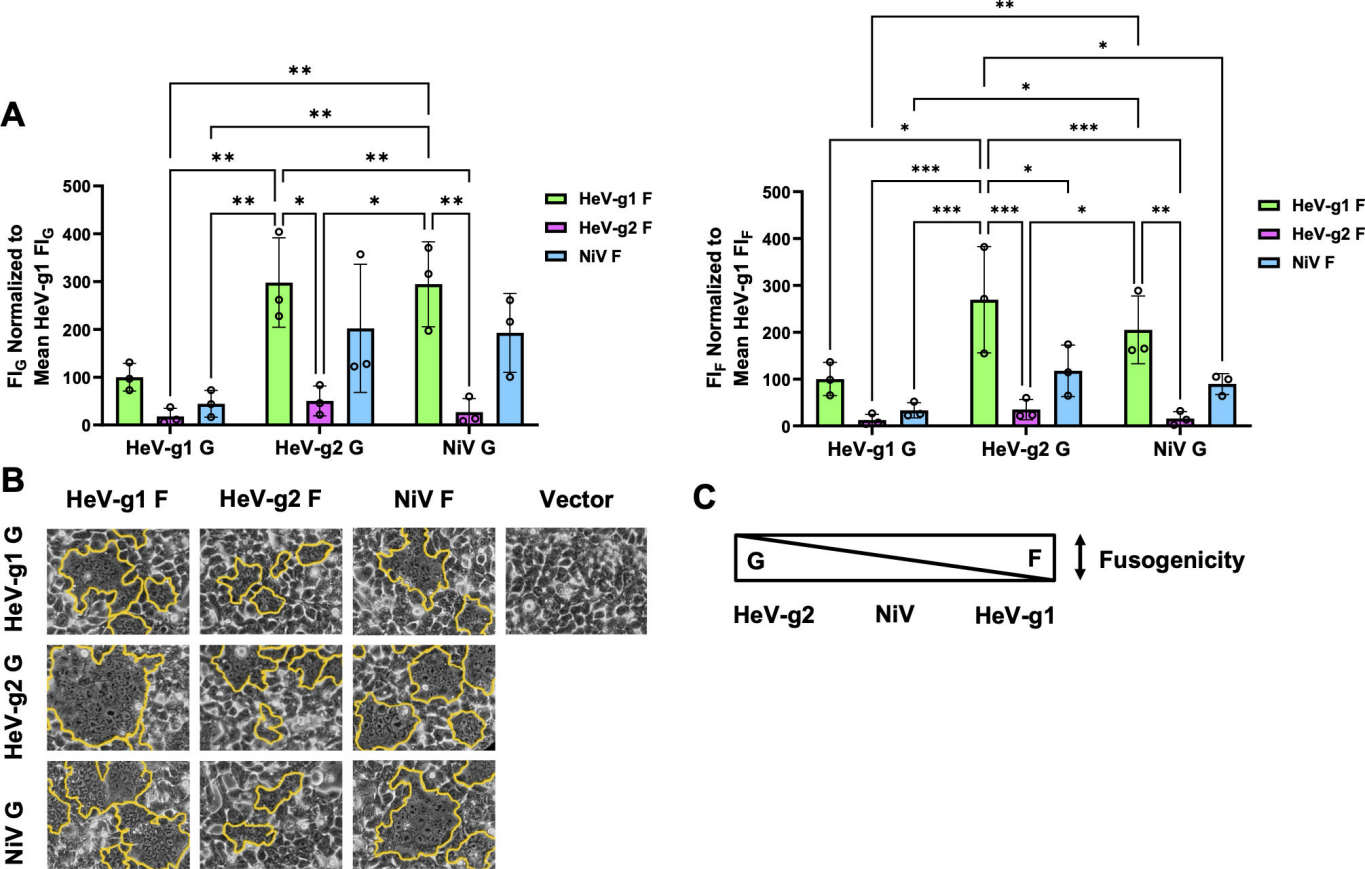
Cell-cell fusion phenotypes using heterotypic combinations of HeV-g1, HeV-g2, and NiV glycoproteins. (**A**) Relative fusion indices normalized to the mean HeV-g1 FIs (calculated as described in Fig. 3). (**B**) Representative syncytia images in HEK 293T cells. (**C**) Schematic of relative intrinsic fusogenicities of HNV glycoproteins, where increasing bar width corresponds to increased fusion levels. Statistical significance was determined via two-way analysis of variance with Tukey’s multiple comparison test. Only significant *P* values are shown with the following indicators: *P ≤ 0.05, **P ≤ 0.01, ***P ≤ 0.001.

Based on the fusion indices obtained when normalized to G_CSE_ levels (FI_G_), HeV-g2 G conferred levels of cell-cell fusion higher than HeV-g1 G (consistent with our results in Fig. 3) but comparable to NiV G (Fig. 4A). Meanwhile, based on the fusion indices obtained when normalized to F CSE levels (FI_F_), HeV-g2 F conferred levels of cell-cell fusion lower than both HeV-g1 F (also consistent with our results in Fig. 3) and NiV F (Fig. 4A). The fusogenic capacities of NiV glycoproteins may also be more similar to HeV-g2 than HeV-g1 (Fig. 4A). Taking these trends altogether, we found that HeV-g2 G appears to exhibit slightly more fusion-promoting activity than NiV G, but HeV-g1 G exhibits the least fusion-promoting activity, whereas HeV-g2 F is consistently the least fusogenic among the F proteins. Representative images are included in Fig. 4B, and the relative fusogenic capacities of these glycoproteins are summarized in Fig. 4C.

### HeV glycoprotein chimeras reveal the ability for multiple glycoprotein domains to modulate HeV fusogenicity

**Fig 5:**
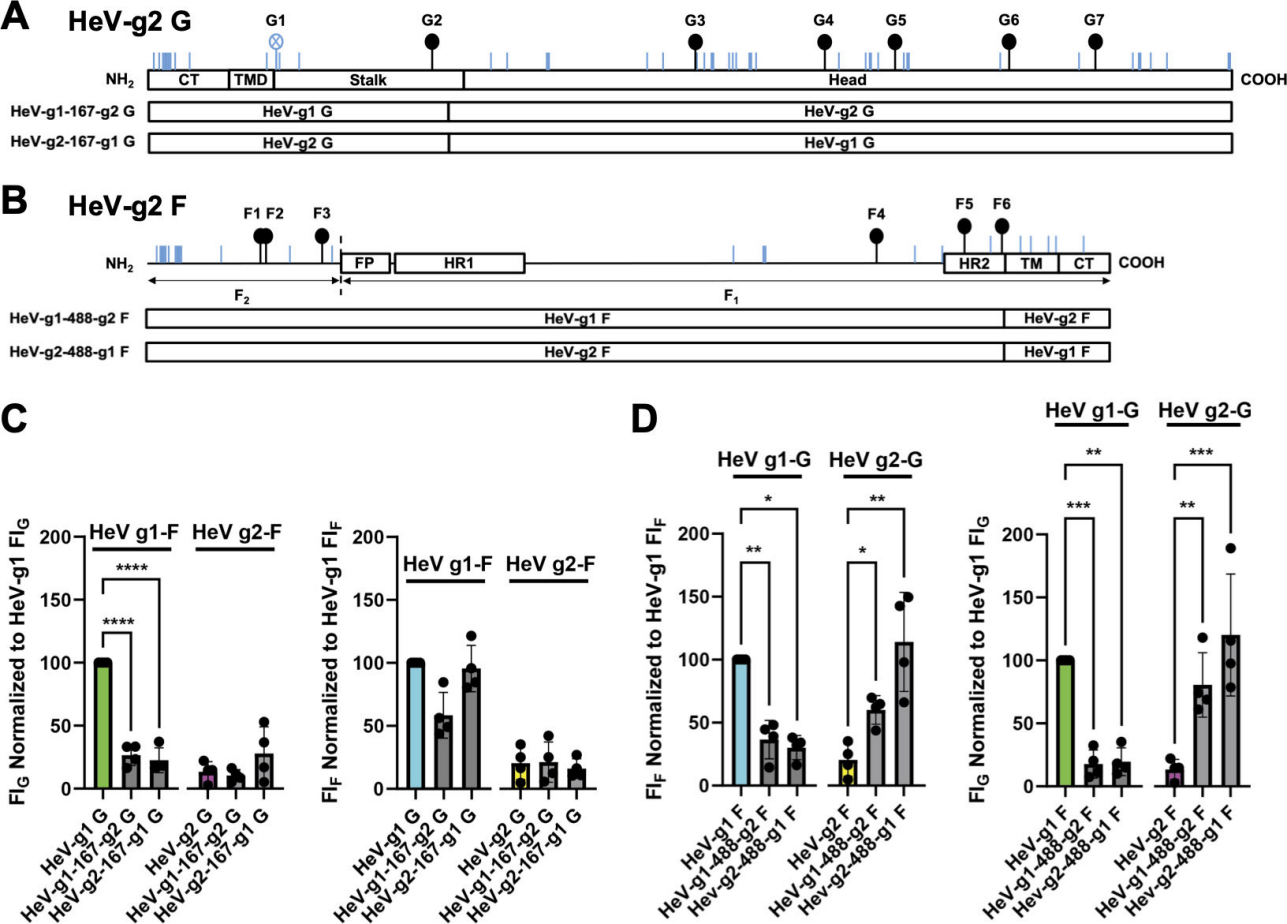
Cell-cell fusion activities of HeV glycoprotein chimeras. (**A and B**) Schematics of the chimeric HeV glycoproteins. Chimeric HeV G glycoproteins were constructed by swapping the head domains of HeV-g1 G and HeV-g2 G, yielding the HeV-g1-167-g2 G and HeV-g2-167-g1 G chimeras. Chimeric HeV F glycoproteins were constructed by swapping the transmembrane (TM) and cytoplasmic tail (CT) domains of HeV-g1 F and HeV-g2 F, yielding the HeV-g1-488-g2 F and HeV-g2-488-g1 F chimeras. (**C**) Relative FI_G_ normalized to HeV-g1 FI_G_ and relative FI_F_ normalized to HeV-g1 FI_F_ across HeV G chimeras. (**D**) Relative FI_G_ normalized to HeV-g1 FI_G_ and relative FI_F_ normalized to HeV-g1 FI_F_ across HeV F chimeras. Statistical significance was determined via two-way analysis of variance with Tukey’s multiple comparison test. Only significant *P* values are shown with the following indicators: *P ≤ 0.05, **P ≤ 0.01, ***P ≤ 0.001, ****P ≤ 0.0001.

In an attempt to map the domains of HeV-g2 glycoproteins that modulate cell-cell fusion, we generated chimeric proteins by swapping the head vs other domains of HeV-g1 G and HeV-g2 G, or the ectodomain vs transmembrane (TM) and CT domains of HeV-g1 F and HeV-g2 F (Fig. 5A and B), which include the key fusion-regulatory domains we previously identified (30, 31, 35). Our results revealed that although HeV-g2 G consistently promoted high levels of fusion (Fig. 4A), both G chimeras promoted lower levels of fusion when co-transfected with HeV-g1 F but induced similar levels of fusion to native HeV-g2 G when co-transfected with HeV-g2 F (Fig. 5C). This suggests that both the head domain and the stalk/TM/CT domains of the G protein are involved in fusion promotion.

Similarly, although HeV-g1 F was the most hyperfusogenic F protein (Fig. 4B), we found that including the TM/CT domains of HeV-g1 F produced variable levels of cell-cell fusion (Fig. 5D). We also found no significant differences in fusogenicity between the HeV F chimeras regardless of context (Fig. 5D). Interestingly, both the F chimeric proteins produced significantly lower fusion levels than wild-type HeV-g1 F when paired with HeV-g2 G. In contrast, the F chimeras yielded higher fusion levels than wild-type HeV-g2 F when paired with HeV-g2 G. This suggests that fusion phenotypes of the F protein may be coordinated through the concerted interactions across multiple domains. For example, we have shown that bidentate interactions between F and G glycoproteins are important for NiV fusion (29).

## DISCUSSION

Pathogenic zoonotic viruses, such as the HNVs, are of considerable importance due to their risk of spillover infections and disease (2, 36). Although HeV-g2 has been detected in two fatally diseased horses and in *Pteropus* spp. or fruit bats (24–27), its use of the highly conserved ephrinB2 and B3 as entry receptors supports its potential to infect humans, as well as other mammalian species, including pigs, cats, ferrets, guinea pigs, and hamsters (8). Furthermore, we showed that HeV-g2 glycoproteins are capable of mediating syncytia formation in human cells, which suggests that HeV-g2 could infect humans.

The conserved features between HeV-g2 and HeV-g1 glycoproteins illuminate important aspects retained in HNVs. For instance, the HeV-g2 G stalk domain is well conserved (Fig. 1D), which is consistent with the relatively conserved stalk C-terminus across HNV Gs (31). This is congruent with the central role of the HNV G stalk in viral entry, as it has been shown that exposure of the stalk C-terminus triggers F to mediate membrane fusion (31, 37, 38). In addition, mutations along NiV G and HeV-g1 G stalks have produced numerous fusion-defective phenotypes (38–40), suggesting that HNV G stalks are indispensable and may have a constrained mutational landscape. Most mutations of HeV-g2 G were found on its head domain (28), a feature consistent with studies on HNV G head domains that revealed their structural plasticity (9–11, 13–17, 19). Meanwhile, HeV-g2 F remains largely conserved (Fig. 1E), an observation consistent with the relatively more conserved functions of paramyxoviral fusion proteins (19). Within the F_2_ subunit, a high proportion of mutations are concentrated near the N-terminus (Fig. 1E), but these mutations occur away from a conserved region within the F_2_ subunit that plays a role in regulating membrane fusion (41). Therefore, should emerging HNV variants continue to retain conserved G stalks and F proteins, these regions may be promising targets for broad-spectrum HNV vaccines and antiviral therapeutics.

Despite HeV-g1 and HeV-g2 sharing high-sequence conservation (Fig. 1A), G oligomeric states, F cleavage (Fig. 2C), receptor tropism (Fig. 2D and E), and antigenic profiles (Fig. 2F), we found that HeV-g2 exhibited significantly hypofusogenic activity relative to HeV-g1 (Fig. 3) due to contrasting fusogenic capacities of their G and F glycoproteins (Fig. 4). The divergence in HeV-g2 G and F’s intrinsic capacities to induce cell-cell fusion supports our hypothesis that both G and F are important modulators of the paramyxovirus membrane fusion process, at least during syncytia formation (22). Moreover, as HeV-g2 F displays a drastically hypofusogenic phenotype that is only partially ameliorated by the hyperfusogenic phenotype of HeV-g2 G (Fig. 3), a balance in promoting cell-cell fusion between the two glycoproteins may also be required to achieve viral fitness. Interestingly, such fusogenic capacities appear to be more similar between HeV-g2 and NiV, with both their G glycoproteins displaying high fusion-promotion activity and both their F proteins displaying hypofusogenic activity relative to HeV-g1 (Fig. 4).

The consistently low fusion-promoting phenotypes of the HeV G head/stalk chimeras (Fig. 5C) align with our previous study (30), where NiV and HeV-g1 G chimeras similarly yielded consistently low fusion-promoting phenotypes despite NiV G inducing higher levels of cell-cell fusion than HeV-g1 G (Fig. 4C). These data suggest that the HNV G head and stalk domains are functionally dependent on each other, which is in agreement with our previous studies highlighting the importance of head and stalk bidentate interactions between HNV G and F for mediating cell-cell fusion (29–31).

Although accumulating evidence suggests a possible correlation between HNV fusogenicity and pathogenicity (8, 16, 31), both HeV-g1 and HeV-g2 infections in horses have resulted in mortality and zoonosis (24–27) despite the significantly hypofusogenic phenotype of HeV-g2 (Fig. 3 and 4). This suggests that other viral proteins, including the immunosuppressive P, V, and W viral proteins, may also contrast in their functional capacities and play important roles in HeV-g2’s retained pathogenicity. The disparity in the fusogenic activities of heterotypic HeV-g1 and HeV-g2 glycoprotein pairs may also have important implications on HeV disease outcomes, as both HeV genotypes have been detected in tissues from *Pteropus poliocephalus* and *Pteropus scapulatus*, as well as in urine excreted by *Pteropus alecto* reservoir hosts. Although no co-infections have been reported in large screening efforts in these flying fox species or spillover hosts (horses) in overlapping geographical areas, this is confounded by the fact that their glycoproteins appear to be serologically indistinguishable (24–27) (Fig. 2G). This suggests other drivers of evolution besides antibody-mediated antigenic drift.

Overall, our study supports the effectiveness of characterizing conserved and deviating features of emerging pathogenic HNVs by performing a side-by-side comparison to closely related HNVs. This strategy may help predict important characteristics of future HNV strains and species, such as their evolution, pathogenicity, host susceptibility, spillover potential, and vaccination and antiviral strategies, altogether assisting our continued efforts in studying HNV glycoproteins and combating future emerging HNVs.

## MATERIALS AND METHODS

### Protein sequence accession numbers

The accession numbers of the protein sequences used in this study are as follows: NiV-B: AEZ01396.1 and AEZ01397.1; NiV-M: AAK50553.1 and APT69701.1; HeV-g1: APT69529.1 and NP_047112.2; HeV-g2: QYC64604.1 and QYC64605.1; CedV: YP_009094085.1 and YP_009094086.1; and MojV: YP_009094094.1 and YP_009094095.1.

### Phylogenetic alignments

All sequences were downloaded from National Center for Biotechnology Information’s (NCBI) GenBank protein sequences collection via the accession numbers listed above. Amino acid sequences were then aligned with multiple sequence comparison by log-expectation (MUSCLE) (42, 43) using a maximum of 16 iterations and curated via Gblocks using stringent parameters. Maximum likelihood phylogenies were generated via PhyML (44) and rendered with TreeDyn (45) using a bootstrap value of 500. FASTA sequences with the corresponding accession numbers above were downloaded from NCBI’s GenBank protein sequence collection.

### Expression plasmids

HeV-g2, HeV-g1, CedV, NiV, and HeV chimeric glycoprotein genes were codon-optimized and cloned between KpnI and XhoI restriction sites in pCAGGS or pcDNA3.1 expression vectors. G genes contained an HA tag at the C-terminus, and F genes contained an internal FLAG tag at identical locations as described previously (24, 25, 31).

### Cell lines

HEK 293T cells were maintained at 37°C and 5% CO_2_ in Dulbecco’s modified Eagle’s medium (DMEM) with 10% bovine calf serum (BCS) and 1% pen-strep.

### Cell transfection

Cells at around 80% confluency were transfected with G and F DNA expression plasmids. Backbone plasmids were used as negative control. In single glycoprotein transfections, 3-µg DNA in 1 mL of OptiMEM was added following 15 min of incubation with 12 µg polyethylenimine at room temperature. After 4 hours at 37°C, 1 mL of complete DMEM was added per well, and the cells were collected 12–16 hours post-transfection. In homotypic and heterotypic glycoprotein transfections, the same amounts of reagents were used. The 3 µg DNA was distributed between the G and F plasmids to obtain comparable G_CSE_ and F_CSE_ across all experimental replicates, and cells were then collected 8 hours post-transfection.

### Immunoblotting

Transfected cells were pelleted and lysed in 1× Triton X-100 radioimmunoprecipitation assay buffer (200 µL) with occasional vortexing. Cell lysates (30 µL) were loaded with 1× loading dye containing 5% beta-mercaptoethanol (excluded for non-reducing gels) and heated at 95°C. Cell lysates were separated by 10% SDS-PAGE under 100 V for 100 min, tank-transferred to polyvinylidene fluoride membranes under 0.5 A for 100 min, blocked with LI-COR Odyssey Blocking Buffer, and stained with primary (Invitrogen mouse anti-FLAG or rabbit anti-HA) and secondary (BioLegend anti-mouse 647 or anti-rabbit 488) antibodies. Bands were detected using a ChemiDoc MP Imager system, and densitometric analyses were performed on Bio-Rad Image Lab.

### Flow cytometry

Transfected cells were resuspended and transferred to 96-well plates on ice. All antibodies were added at 50 µL/well. For ephrin binding, the primary staining was performed with one of the soluble recombinant mouse ephrinB1/human Fc, ephrinB2/human Fc, or ephrinB3/mouse Fc chimeric proteins obtained from R&D Systems (100 nM), and the secondary staining was performed with the corresponding anti-human or anti-mouse Alexa Fluor 647 antibodies obtained from Invitrogen (1:1,000). Primary staining was incubated for 1 h on ice, followed by secondary staining for 30 min on ice. For CSE, stainings were done with primary conjugated Alexa Fluor 647 mouse anti-HA or rat anti-FLAG antibodies obtained from BioLegend (1:1,000). For cross-reactivity, the primary staining was performed with polyclonal rabbit HeV-g1 antisera (1:300) (33), and the secondary staining was performed with anti-rabbit Alexa Fluor 647 antibodies (1:1,000). All intermediate wash steps were performed with phosphate-buffered saline with 1% BCS. Stained cells were analyzed by flow cytometry (Guava easyCyte8 HT).

### Cell imaging

Cells were imaged via the ECHO Revolve at ×200, bright field. Cells were imaged at room temperature immediately after 8 hours post-transfection.

### Syncytia assays

The number of nuclei inside multinucleated cells (syncytia) were manually counted under ×200 magnification from five microscopic fields, as performed in previous studies. To avoid cell division to be confused with syncytia, only syncytia with ≥4 nuclei were counted, as in prior studies (22, 32, 33).

### Statistical analyses

All comparisons were made via pairwise *t*-tests or two-way analysis of variance with Tukey’s multiple comparison test in Prism. All error bars represent standard deviation. All experiments were completed with *n* = 3 biological replicates.
